# Adapting evidence-informed complex population health interventions for new contexts: a systematic review of guidance

**DOI:** 10.1186/s13012-019-0956-5

**Published:** 2019-12-17

**Authors:** A. Movsisyan, L. Arnold, R. Evans, B. Hallingberg, G. Moore, A. O’Cathain, L. M. Pfadenhauer, J. Segrott, E. Rehfuess

**Affiliations:** 1 Institute for Medical Information Processing, Biometry and Epidemiology, LMU Munich, Marchioninistrasse 17, 81377 Munich, Germany; 2 Pettenkofer School of Public Health, LMU Munich, Marchioninistrasse 17, 81377 Munich, Germany; 3 Centre for the Development and Evaluation of Complex Interventions for Public Health Improvement (DECIPHer), School of Social Sciences, Cardiff University, 1-3 Museum Place, CF10 3BD Cardiff, Wales UK; 4 Cardiff School of Sport & Health Sciences, Llandaff Campus, Cardiff Metropolitan University, Western Avenue, Cardiff, CF5 2YB Wales UK; 5 School of Health and Related Research (ScHARR), University of Sheffield, Regent Court, 20 Regent Street, S1 4DA Sheffield, UK

**Keywords:** Adaptation, Replication, Guidance, Implementation, Context, Evidence-based, Evidence-informed, Systematic review, Population health, Complex interventions

## Abstract

**Background:**

Adapting interventions that have worked elsewhere can save resources associated with developing new interventions for each specific context. While a developing body of evidence shows benefits of adapted interventions compared with interventions transported without adaptation, there are also examples of interventions which have been extensively adapted, yet have not worked in the new context. Decisions on when, to what extent, and how to adapt interventions therefore are not straightforward, particularly when conceptualising intervention effects as contingent upon contextual interactions in complex systems. No guidance currently addresses these questions comprehensively. To inform development of an overarching guidance on adaptation of complex population health interventions, this systematic review synthesises the content of the existing guidance papers.

**Methods:**

We searched for papers published between January 2000 and October 2018 in 7 bibliographic databases. We used citation tracking and contacted authors and experts to locate further papers. We double screened all the identified records. We extracted data into the following categories: descriptive information, key concepts and definitions, rationale for adaptation, aspects of adaptation, process of adaptation, evaluating and reporting adapted interventions. Data extraction was conducted independently by two reviewers, and retrieved data were synthesised thematically within pre-specified and emergent categories.

**Results:**

We retrieved 6694 unique records. Thirty-eight papers were included in the review representing 35 sources of guidance. Most papers were developed in the USA in the context of implementing evidence-informed interventions among different population groups within the country, such as minority populations. We found much agreement on how the papers defined key concepts, aims, and procedures of adaptation, including involvement of key stakeholders, but also identified gaps in scope, conceptualisation, and operationalisation in several categories.

**Conclusions:**

Our review found limitations that should be addressed in future guidance on adaptation**.** Specifically, future guidance needs to be reflective of adaptations in the context of transferring interventions across countries, including macro- (e.g. national-) level interventions, better theorise the role of intervention mechanisms and contextual interactions in the replicability of effects and accordingly conceptualise key concepts, such as fidelity to intervention functions, and finally, suggest evidence-informed strategies for adaptation re-evaluation and reporting.

**Trial registration:**

PROSPERO 2018, CRD42018112714.

Contributions to the literature

- Making decisions about interventions to improve population health often relies on evidence from a different context.
- To replicate the effects observed in the context in which interventions were developed and tested, these may need to be adapted for a given new context.
- Differences between contexts may introduce uncertainty warranting re-evaluation in the new context.
- This systematic review synthesises definitions of key concepts and recommendations on undertaking adaptations that support implementation, and evaluating the adapted intervention in a new context.
- Our review provides a state-of-the-art catalogue of existing guidance and identifies limitations to inform development of an overarching guidance on adaptation.

## Background

Population health interventions comprise a spectrum of interventions, programmes, and policies in public health and health services research that seek to change the population distribution of risk [1]. This includes interventions delivered to whole populations (e.g. regulatory restrictions on alcohol sales), and interventions targeting defined sub-populations (e.g. based on age), or specific groups with increased levels of risk (e.g. brief alcohol interventions for harmful drinkers or health service interventions to prevent obesity [1]). Increasingly, interventions are seen as interacting with the complex systems into which they are introduced [2–4]. From this systems perspective, all interventions can be conceptualised as complex, as they operate through active contextual interactions, influence and are influenced by mechanisms of the entire system [3].

Implementing interventions that have worked elsewhere (we refer to these as evidence-informed interventions) can save human and financial resources associated with building evidence de novo for each context. However, this often involves implementing an intervention in systems with different norms, resources, and delivery structures to the original context. While there are examples of complex population health interventions that have successfully been transferred to new contexts [5, 6], others have been ineffective [7, 8], or even harmful [9, 10]. Potential reasons for transferability failure include contextual disparities, local adaptations which compromise important intervention functions, or different evaluation methods in the original and new contexts [11].

Context can be thought of as a set of characteristics and circumstances that consist of active and unique factors within which the implementation of an intervention is embedded [12]. Intervention effects are generated through interaction of new ways of working with existing contexts [13]. When implementing interventions in a new context, adaptation and re-evaluation is often required to be confident that the intervention will achieve the same benefits as in the original study. Simultaneous recognition of the value of using evidence from elsewhere, and the need to adapt interventions to achieve fit within new contexts, has stimulated research on the implementation and/or re-evaluation of evidence-informed interventions in new contexts.

A number of papers, including editorials and case studies, have been published in recent years providing recommendations on how to adapt interventions for new contexts [11, 14]. However, few attempts have been made to systematise them [15], and no overarching and consensus-based guidance is currently available. Furthermore, there are debates in the field on how to define and operationalise important concepts, such as adaptation and fidelity. For example, Stirman et al. define adaptations based on the targets of modification [16]: (i) modifications made to the content of the intervention and its implementation; (ii) modifications made to the context; and (iii) modifications made to procedures for intervention evaluation [16]. In the meantime, Resnicow et al. suggest defining adaptations based on degrees of modification: modifications made to observable characteristics of the intervention (i.e. surface structures) and those made to the underlying psycho-social and environmental factors (i.e. deep structures). Different approaches have also been put forward regarding “fidelity”. Implementation fidelity has commonly been conceptualised as delivery of a (manualised) intervention as intended by developers [17]. Proponents of complex systems thinking, however, have suggested alternatively defining fidelity as retaining important functions (i.e. the mechanisms and theoretical principles) of the intervention while allowing adaptations to form (i.e. specific content and delivery) of the intervention [18].

The ADAPT Study has been funded to develop an evidence-informed and consensus-based guidance for adapting complex population health interventions to new contexts [11, 19]. Commensurate with best practices in guidance development [20], the ADAPT Study follows a phased process, incorporating existing methodological knowledge through literature reviews and expert consultations, as well as the use of consensus development methods. A comprehensive literature review serves to consolidate existing knowledge on the topic, notably the spectrum of necessary considerations, and to identify relevant stakeholder groups to consult. This systematic review has thus been designed as the first stage of a broader guidance development to synthesise existing recommendations on adaptation and inform the further phases of the study, including qualitative interviews with key stakeholders and an international Delphi panel [11].

We found only one recent scoping review of adaptation frameworks in public health [15], which maps existing recommendations on adaptation. However, the review only focuses on key steps described in the frameworks and does not provide in-depth analysis of important concepts and strategies in adaptation or assess approaches to intervention re-evaluation in new contexts; nor does it extend to health services research. To address this gap, the present systematic review aims to provide a comprehensive synthesis of existing guidance on intervention adaptation in relation to (i) key concepts, (ii) the rationale for adaptation, (iii) different types of adaptations, (iv) the processes recommended for conducting intervention adaptation, and (v) methodological approaches suggested to re-evaluate and (vi) report the adapted intervention.

## Methods

The systematic review was conducted in accordance with the Preferred Reporting Items for Systematic Reviews and Meta-Analyses (PRISMA) guidelines (see Additional file 1 for completed PRISMA checklist) [21]. The review protocol was pre-registered in PROSPERO (CRD42018112714) and the Open Science Framework (osf.io/wn5f8).

### Search strategy

We designed the search strategy iteratively in consultation with information specialists to achieve balance between sensitivity and specificity so that the search would retrieve all pre-identified eligible studies and yield a manageable number of studies to screen. We ran the searches on October 12, 2018 in the following databases: Applied Social Sciences Index & Abstracts (ASSIA), Conference Proceedings Citation Index – Social Science & Humanities (CPCI-SSH), Dissertations and Theses Global: The Humanities and Social Sciences Collection, EMBASE, MEDLINE and Epub Ahead of Print, In-Process & Other Non-Indexed Citations, Daily and Versions, PsycINFO, and Social Science Citation Index (SSCI). We used citation tracking (backward and forward in Google Scholar) of all included studies and contacted authors and international experts to locate further studies and updates (see Additional file 2 for the search strategy).

### Eligibility criteria

To be included, a document had to be full-length and provide recommendations on how to adapt and/or re-evaluate interventions in new contexts. We define full-length documents as those with substantive narrative, such as research, analysis, methodological papers, or dissertations, theses, and book chapters. We did not consider commentaries, abstracts, information available on web-pages, and conference proceedings without a link to a full report as full-length documents. To include a range of perspectives, we did not limit the review scope to only those papers that described a formal process of guidance development, but rather included all papers that described recommendations for practice. Papers providing only conceptual discussion on or examples of intervention adaptation without explicit recommendations for practice were not included; these were saved in a separate category during data screening for consideration in a related scoping review on “cases of adaptation” (Open Science Framework registration: osf.io/udzma). Further inclusion criteria were as follows: (i) focus on public health and/or health service interventions rather than on specific clinical procedures, such as surgery, (ii) publication from 2000 onwards, as this was when discussions on evidence-informed interventions and their adaptation came to the fore [15, 22], (iii) publication in English, German, French, Italian, Russian, or Swedish, as these languages could be comprehensively covered by the project team members. Table 1 provides further clarifications for the eligibility criteria.

**Table 1:** Eligibility criteria

Criterion	Definition
Guidance	● Full-length document providing advice and specific recommendations on key concepts and/or steps, principles, and strategies for adapting population health interventions in new contexts*.* ● The included documents may be intended for researchers, practitioners, and/or funders to support in their use of methods and conduct of research on intervention adaptation.
Document type	● Peer-reviewed papers: research, analysis, or methodological papers. ● Non peer-reviewed documents: dissertations, theses, books, book chapters, governmental and non-governmental reports, and working papers (i.e. documents issued by local, regional, or national governments or by their agencies or subdivisions, as well as those written and published by non-governmental organisations).
Adaptation	● Modifications made to the content of interventions and their implementation AND/OR ● Modifications made to the context in which interventions are delivered AND/OR ● Modifications made during the evaluation processes
New context	● Characterised by differences in geographical, epidemiological, socio-cultural, socio-economic, ethical, legal, and/or political determinants. NB: Guidance papers which describe scale-up of interventions will be included only if the scale-up is described with regard to changes in any of the above-mentioned determinants (e.g. taking interventions tested in a specific geographical district for full-scale implementation in other districts, which differ in their contextual profile, such as population and socio-economic determinants).
Population health interventions	● Interventions, programmes, and policies which seek to change the population distribution of risk. ● These interventions can be delivered to whole populations, defined sub-populations (based on age or other characteristic), or specific groups with increased levels of risk. ● Interventions may encompass public health or health services research.
Language	● Papers written in English, German, French, Italian, Russian, or Swedish.
Geographical location	● Any

### Study selection

Results were imported into the Endnote reference management software and de-duplicated. One reviewer (AM) screened publications on title level and removed clearly irrelevant retrievals using the eligibility criteria in Table 1. Two reviewers (shared among AM, BH, LA, and LP) independently screened the titles and abstracts of the remaining records followed by full-text screening (AM and LA). Disagreements or uncertainties regarding eligibility were resolved by discussion among the two reviewers, with recourse to a third when necessary (RE). Data screening was performed using the Rayyan web application for systematic reviews [23].

### Extraction

We developed the data extraction form based on the review objectives (see Additional file 3). The initial form was piloted by four reviewers (AM, ER, LA, and RE) on two eligible papers. Uncertainties during piloting were noted and discussed among the four reviewers to revise and finalise the form. Two reviewers (AM and LA) then independently extracted all data onto seven pre-specified categories:

1. Descriptive information including publication author, year, title, and source.
2. Key concepts of adaptation used, including employed definitions and nomenclature.
3. Rationale for intervention adaptation, including why and when adaptations should be undertaken.
4. Types and components of adaptation
5. Processes for undertaking adaptation
6. Approaches for deciding on an appropriate methodology for re-evaluating the adapted intervention.
7. Suggested criteria or recommendations on how to report intervention adaptations.

Disagreements and ambiguities regarding the extraction were resolved by discussion among the two reviewers.

### Synthesis

We synthesised extracted data using procedures derived from thematic and cross-case analyses, such as descriptive coding and cross-case tabulation [24–26]. Thematic analysis is widely used for analysing textual data, and in combination with cross-case analysis facilitated examination of commonalities and differences of the content of the guidance papers in this review [26]. First, we used the seven pre-specified categories described above to sort the data. To do this, we employed structural coding described by Saldaña [24], which applies a content-based phrase representing a topic of inquiry to large segments of data relating to a specific research question (e.g. rationale for adaptation). Drawing on the cross-case analytical approach described by Miles and Huberman [25], two reviewers (AM and LA) charted the data to examine how data in each category were described across the papers (e.g. how different papers described the rationale for adaptation). For this, we employed a more inductive and descriptive line-by-line coding. Synthesis drafts and descriptions of each category were then developed by two reviewers (AM and LA), examined by all authors, and revised based on their feedback.

### Quality appraisal

We assessed included papers against pre-defined criteria designed by the project team drawing on related previous work [12, 27, 28]. While papers were not excluded on the grounds of this assessment, we assigned more interpretive weight to those with clearer concepts and more comprehensive guidance. Included papers were assessed against three criteria, namely *practicality* (defined as understandability and clarity of key constructs, ease of use and comprehensiveness in terms of coverage of adaptation and evaluation recommendations), *relevance* (defined in terms of applicability to different types of interventions and by different stakeholder groups, such as researchers and funders), and *legitimacy* (defined as following a “formal process” of guidance development, such as using a literature review and/or a consensus-based methodology). We assigned a rating of 0 (criterion is not addressed at all), + (criterion is partially addressed), or ++ (criterion is fully addressed). Two reviewers independently conducted appraisals (AM and LA) and resolved any disagreements through discussion (see Additional file 4 for further details on the criteria).

## Results

**Fig. 1:**
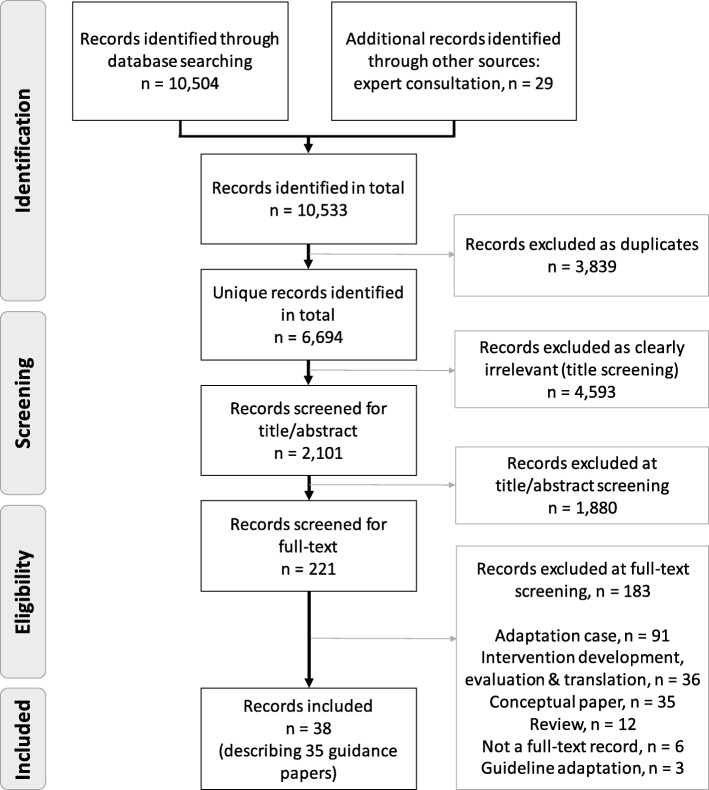
Systematic review flow diagram

Our database searches identified 6694 unique records of which 38 records were included in the review describing 35 guidance papers (see Fig. 1 for the PRISMA flow diagram). No additional papers were found based on citation tracking or expert recommendations.

### Characteristics of included studies

As shown in Table 2, papers varied in their topic area and largely focused on sexual health and HIV/AIDS prevention programmes (*n* = 8, 23%), parenting and family-based interventions (*n* = 8, 23%), and psychotherapies (*n* = 6, 17%). Thirty-one papers (89%) described and drew on micro-level interventions (i.e. those that focus on intervening with individuals and their immediate social network and relationships, such as the family) to illustrate the application of the guidance. Meso-level interventions (i.e. those that focus on intervening with population groups, such as neighbourhoods, schools, or other community organisations) were discussed in five (14%). We did not identify a paper that discussed adaptation of macro-level interventions (i.e. those that focus on intervening with overarching social systems that operate at the national or global level). Based on the affiliation of the first author, 28 papers (80%) were developed in the USA and 24 of these discussed adaptations across different populations within the USA (e.g. transferring interventions to ethnic minority groups within the USA) [29–52]. Most papers were published in peer-reviewed journals (*n* = 31, 89%) [29–31, 33–36, 38–61]; we identified 4 papers (11%) from grey literature sources, including two book chapters [32, 37] and two governmental agency reports [62, 63].

**Table 2:** Characteristics of the included guidance papers

First author (year)	Short title/name of guidance	Topic area of guidance	Level of intervention^a^	Theoretical principles	Country of origin	Stepwise approach
Aarons (2012) [29]	Dynamic adaptation process (DAP)	Child neglect	Micro	Not specified	California, USA	Yes
Aarons (2017) [30]	“Scaling-out” EBIs	HIV/AIDS, family-based parenting	Micro	Cook’s five pragmatic principles	California, USA	No
Backer (2002) [62]	Finding the balance between programme fidelity \and adaptation	Substance abuse	Not specified	Roger’s diffusion of innovations theory	Washington DC, USA	Yes
Barrera (2006) [31]	A heuristic framework for cultural adaptation	Parenting, psychotherapy	Micro	Not specified	Arizona, USA	Yes
Bartholomew (2016) [32]	Using intervention mapping (IM) to adapt EBIs	Breast cancer screening	Micro	Not specified	Houston, USA	Yes
Bernal (2006) [33]	Culturally centred psycho-social interventions	Psychotherapy	Micro	Not specified	Puerto Rico, USA	No
Card (2011) [34]	How to adapt effective programmes for use in new contexts	HIV/AIDS (one-on-one and community-based)	Micro, meso	Not specified	Los Altos, USA	Yes
Cardemil (2010) [35]	Cultural adaptations to empirically supported treatments	Psychotherapy	Micro	Not specified	Worchester, USA	No
Chen (2012) [36]	Programme adaptation through community engagement	Arthritis self-help	Micro	CBPR	Ithaca, USA	Yes
Davidson (2013) [53]	A tool kit of adaptation approaches	Behaviour change interventions	Micro	Not specified	Edinburgh, UK	Yes
Domenech-Rodriguez (2005) [37]	Culturally appropriate EBTs for ethnic minority populations	Parenting, psychotherapy	Micro	Roger’s diffusion of innovations theory	Utah, USA	Yes
Goldstein (2012) [38]	Guidelines for adapting manualised interventions for new populations	Anger management	Micro	Participatory Action Research (PAR)	Philadelphia, USA	Yes
Hwang (2006) [39]	The psychotherapy adaptation and modification framework (PAMF)	Psychotherapy	Micro	Top-down theoretical approach	Claremont, USA	No
Hwang (2009) [40]	The Formative Method for Adapting Psychotherapy (FMAP)	Psychotherapy	Micro	Bottom-up theoretical approach	Claremont, USA	Yes
Kemp (2016) [54]	Adaptation and fidelity: a recipe analogy	Nurse home visiting	Micro	Not specified	Sydney, Australia	No
Kilbourne (2007) [41]	Application of the Replicating Effective Programmes (REP) framework	Psycho-education, HIV/AIDS	Micro	Roger’s diffusion of innovations theory	Michigan, USA	Yes
Kumpfer (2008–2016) [55, 64, 66]	Cultural adaptation of evidence-based family interventions	Family-based parenting	Micro	CBPR	Utah, USA	Yes
Lau (2006) [42]	Selective and directed cultural adaptations of EBTs	Parenting, psychotherapy	Micro	Not specified	Los Angeles, USA	No
Lee (2008) [43]	Planned adaptation to implement EBPs with new populations	Job-search skill enhancement	Micro	Not specified	Detroit, USA	Yes
Maríñez-Lora (2016) [44]	A framework for translating an EBI from English to Spanish	Family-based parenting	Micro	CBPR	Chicago, USA	No
McKleroy (2006) [45]	Adapting EBIs for new settings and target populations	HIV/AIDS	Micro	Roger’s diffusion of innovations theory; CBPR	Atlanta, USA	Yes
Nápoles (2013) [46]	Methods for translating EBIs for health-disparity communities	Behaviour change interventions	Micro	Not specified	Atlanta, USA	Yes
Nápoles (2018) [47]	Transcreation: an implementation science framework	Health disparities	Meso	CBPR	Bethesda, USA	Yes
NCI RTIPs [63]	Guidelines for choosing and adapting programmes	Cancer	Not specified	Not specified	Bethesda, USA	Yes
Netto (2010) [56]	How to adapt health promotion interventions: five principles	Health promotion (one-on-one and community based)	Micro, meso	Not specified	Edinburgh, UK	Yes
Perez (2016) [57]	A modified theoretical framework to assess implementation fidelity	Empowerment strategies for community involvement	Meso	Roger’s diffusion of innovations theory; CBPR	Havana City, Cuba	Yes
Rolleri (2014) [48]	Adaptation guidance for evidence-based teen pregnancy prevention	STI/HIV (one-on-one)	Micro	Not specified	Bellerose, USA	Yes
Solomon (2006) [49]	Adapting efficacious interventions	HIV/AIDS (one-on-one)	Micro	Not specified	Los Altos, USA	Yes
Sundell (2014) [58]	A model for evaluation empirically supported FBIs in new contexts	Family-based interventions	Micro	Not specified	Stockholm, Sweden	Yes
Tomioka (2013) [50]	A four-step protocol for assuring replication with fidelity	Health promotion for older adults (one-on-one)	Micro	Not specified	Honolulu, USA	Yes
Van Daele (2012) [59]	Empowerment implementation: enhancing fidelity and adaptation	Psychotherapy	Micro	CBPR	Leuven, Belgium	Yes
Wainberg (2007) [60]	A model for adapting EBIs to a new culture	HIV/AIDS (one-on-one)	Micro	CBPR	New York, USA	Yes
Wang-Schweig (2014) [51]	A conceptual framework for cultural adaptation at the deep-structure level	Family-based interventions	Micro	Not specified	Berkeley, USA	Yes
Wingood (2008) [52]	ADAPT-ITT: a method for adapting evidence-based HIV interventions	HIV/AIDS (one-on-one)	Micro	Not specified	Atlanta, USA	Yes
Yong (2016) [61]	Framework for cultural adaptation of preventive health programmes	Vaccination (one-on-one and community outreach)	Micro, Meso	Not specified	Ottawa, Canada	No

*CBPR*, community-based participatory research; *EBI*, evidence-based Intervention; *EBP*, evidence-based Programme; *EBT*, evidence-based treatment; *FBI*, family-based intervention; *STI*, sexually transmitted infections

^a^Micro-level interventions focus on intervening with individuals and their immediate social network and relationships, such as the family. Meso-level interventions focus on intervening with population groups, such as neighbourhoods, schools, or other community. Macro-level interventions focus on intervening with overarching social systems that operate at the national or global level

### Quality of studies

With respect to practicality, we rated 21 papers as providing clear definitions of key constructs and 24 papers as offering a well-operationalised procedure for adapting interventions. The latter primarily involved a sequential stepwise approach. However, we judged 21 papers as only partially addressing the criterion of comprehensiveness (defined as coverage of both intervention adaptation and re-evaluation), as they did not provide thorough guidance on intervention re-evaluation in a new context (see Additional file 4 for detailed ratings).

We judged only six papers as fully addressing relevance; the rest had a specific and narrow focus on individual-level interventions (e.g. psychotherapy and behavioural interventions, see Table 2), and we down-rated their relevance for broader health service and public health interventions, notably policy-level interventions.

Finally, we rated 23 papers as partially addressing legitimacy, as they did not report a transparent and rigorous development process, such as consulting a broader range of stakeholders beyond the immediate author team. The papers, however, frequently reported having conducted a literature review or drawing on theoretical principles to ground their approach—primarily the principles of Roger’s diffusion of innovations theory and community-based participatory research (CBPR) (see Table 2).

### Categories

In the following, we describe the findings of the synthesis undertaken for each of the pre-defined categories. We did not find recommendations on reporting of adapted interventions, so this category has been omitted. During data sorting, we identified a new category of stakeholder involvement in adaptation.

#### Category 1: Key concepts and definitions

Table 3 summarises key concepts and their definitions as used in existing guidance papers. In most cases, papers discussed concepts related to adaptation in the background sections of the guidance by referring to previously published literature and debates [29, 31–34, 36, 43, 45, 49, 51–54, 56–59, 61]. Papers commonly conceptualised *adaptation* as a systematically planned and proactive process of modification with the aim to fit the intervention into a new context and enhance its acceptability [29, 30, 34, 35, 45–49, 51–54, 57, 61, 62, 64]. This approach was contrasted to unplanned modifications, which were seen as undesirable changes happening during intervention implementation in a real-world setting likely to result in “intervention drift” (see Table 3) [29, 32, 36, 45, 54, 62]. Alternative terms to adaptation were suggested in some papers. Specifically, the term *reinvention*, originating from diffusion of innovations theory, was used to describe adaptations occurring at a deeper structure level [57] (see “Category 3: Aspects of adaptation” section below). Nápoles and Stewart used the term *transcreation* to highlight active participation of community partners in the process [47].

**Table 3:** Key concepts and definitions

Key concept	Definition
Adaptation	A systematically planned and proactive process of intervention modification [29, 30, 34, 35, 45, 47, 48, 51–54, 57, 62, 64] with the aim to suit the specific characteristics and needs of a new context and enhance intervention acceptability [29, 30, 34, 35, 45, 46, 48, 49, 51, 53, 54, 57, 61, 62, 64]. Mutual adaptation involves adaptation of both the intervention and of the community or organisation in which the intervention is implemented for the purposes of institutional accommodation [57, 62].
Adaptive interventions	Those interventions for which stakeholders are allowed, or even encouraged, to bring changes to the original design. These changes are pre-defined by intervention developers. In the context of complex public health interventions, involving different organisational levels and targeting collective behaviours, implementers can also make changes which are not pre-defined by the developers [31, 57].
Core components	Those features in the intent and design of an intervention which are responsible for the effectiveness of the intervention [32, 34, 36, 41, 43, 45, 48, 49, 52, 62]. Guidance suggests that these components fundamentally define the intervention [34, 43, 45, 48, 62] and therefore should not be modified in adaptation [30, 45, 48, 52], e.g. developing a natural support system for youth and families as part of a family-based intervention. Alternative terms: essential, necessary, prototypical components or elements, or intervention’s deep structure.
Discretionary components	Those features which are not essential for the target audience and which are not supported by the theory of change and thus are assumed to be modifiable without major impact on intervention effectiveness [45, 52, 58, 62], e.g. provision of an additional class as part of a parenting intervention addressing trauma related to natural disasters. Alternative terms: optional components or intervention’s surface structure.
Drift	A misapplication or a mistaken application of an intervention involving technical errors, abandonment of core components, or introduction of counterproductive elements resulting in a loss of intervention benefits [29, 54].
Fidelity (adherence)	The degree to which an intervention is implemented as intended by its developers [29, 47–49, 54, 58, 59, 62] with the aim to maintain intervention’s intended effects [57, 58]. The components of fidelity (also dimensions for measuring fidelity) include dose, frequency, exposure, quality of delivery, participant responsiveness, and programme differentiation [29, 49, 57, 59, 62].
Programme theory	Refers to the causal model that specifies the empirical and theoretical relations between intervention activities, mediators of change, and ultimate outcomes [34, 43, 45, 58]. Alternative terms: theory of change, internal logic
Reinvention	The degree to which an innovation (i.e. an intervention) is changed or modified by the user in the process of its adoption and implementation [37, 57, 62].
Replication	The process of re-implementing an established intervention in a new context in a way that maintains fidelity to core goals, activities, delivery techniques, intensity, and duration of the original study [34].
Transcreation	The processes of planning and delivering interventions so that they resonate with the targeted community, while achieving intended health outcomes [47].
Scale-out	The deliberate use of strategies to implement, test, improve, and sustain an intervention as it is delivered to new populations and/or through new delivery systems that differ from those in effectiveness trials. Aarons et al. distinguish three types of scale-out: type I scale-out: population fixed, different delivery system; type II scale-out: delivery system fixed, different population; type III scale-out: different population and delivery system [30].
Scale-up	The deliberate effort to broaden the delivery of an intervention with the intention of reaching larger numbers of a target audience. It often targets the same or very similar settings, under which the intervention has already been tested [30].
Social validity	Refers to perceived acceptability, utility, and viability of the intervention. These perceptions might be influenced by cultural worldview and the practical realities of life circumstances (e.g. transportation, insurance coverage, and work schedules) [31, 42].

Adaptation and *fidelity* were commonly viewed as mutually exclusive concepts. Sometimes, as remarked by Perez et al., this paralleled a distinction between imposing an intervention on the intended population versus actively engaging with the population to bring about change: “fidelity is underpinned by a professionally driven or “top-down” approach to implementation, while adaptation seems to be closer to a user-based or “bottom-up” approach, which is more politically appealing to promoters of social development” [57]. Resolving the “fidelity-adaption” tension was seen as one of the most challenging tasks in intervention adaptation and was described as a dynamic process that requires strategic revisiting throughout different stages of implementation [62]. In this light, identification of intervention *core components* was highlighted as important in providing a scope for adaptation (see Table 3). Informed by intervention theory, which specifies the theoretical relations between an intervention and its outcomes [34, 45, 58, 62], these components were seen to fundamentally define the intervention [34, 45, 48, 59, 62] and therefore not to be modified during adaptation [30, 45, 48, 52]. In contrast, modification of *discretionary components* was suggested to enhance an intervention’s social validity, that is the perceived acceptability and utility of the intervention [45, 52, 58].

#### Category 2: Rationale and pre-requisites for adapting interventions

Adapting evidence-informed interventions was often described as requiring fewer human and financial resources than newly designing and evaluating interventions in each specific context [39, 40, 45, 48, 52, 55, 58]. As an overarching *aim of adaptation*, the papers highlighted *assuring intervention salience and fit with the new context* and *addressing the specific needs of the local population* [30, 32, 36, 40, 43, 45, 48, 53, 56, 57, 64]. Where detailed, more specific aims of adaptation included enhancing acceptability, local commitment, support, collaboration, and ownership of the intervention [35, 42, 45, 49, 52, 54, 57, 62], facilitating enrolment, engagement, retention, and satisfaction with the intervention [40, 42, 44, 49, 51, 54, 55, 57], as well as supporting successful implementation of the intervention, its use and sustainability [42, 44, 48, 49, 52, 57]. Only a few papers explicitly mentioned maintaining intervention effectiveness as the direct aim of adaptation [49, 51, 53].

To inform the need for specific adaptations, a few *pre-requisite activities* were described, including *exploring the theory underlying the intervention* (also referred to as programme theory and including identification of core components, which should not be modified), *examining the generalisability of intervention effects in multiple contexts* (such as through moderation analysis within randomised controlled trials or other studies), as well as *assessing the extent of mismatch between the candidate and the replication contexts*, and the *acceptability of the intervention in the new context* (see “Category 4: Process of adaptation” on the recommended procedures to assess these) [31, 32, 34, 42, 43, 50, 53, 56, 58]. In most cases, the process of identifying mismatch was described as an assessment of the availability of the resources and infrastructure in the new context (e.g. funding, staffing, and local agency capacity) [34, 45, 48, 49, 58], as well as the distinctive characteristics of the new population (e.g. age, socio-economic status, and cultural norms) [31, 33, 36, 37, 39, 47–49, 56]. While these factors might be linked to intervention theory, none of the papers explicitly emphasised the possible interactions of these contextual factors with intervention mechanisms and implications of these interactions for effects in a new context.

The level of the identified mismatch between the original and new contexts was generally seen to inform the decision about which intervention to select and the extent of adaptations that might be required. As noted by one of the papers: “if mismatches between a candidate programme and a replication context are significant—for example […] if the implementing agency does not have and cannot obtain the resources needed to implement the programme—the programme should probably not be selected for implementation in one’s site. Less significant mismatches may, however, be successfully addressed through the adaptation process” [34]. Exploration of intervention theory was also seen as important in informing the decisions on the degrees of adaptations.

#### Category 3: Aspects of adaptation

Papers discussed different aspects of adaptation. We categorised these in terms of the *targets of adaptation* (i.e. what is modified) and the *degrees of adaptation* (i.e. to what degree).

##### Targets of adaptation

Most frequently, papers discussed *content modifications* as changes including adding, deleting, or changing existing components [33, 35, 43, 44, 46, 54, 57, 64]. While modifications to an intervention’s content were seen to accommodate the needs of the target group, papers cautioned against modifications of “core components”, which were seen as unsafe changes [48, 57]. Papers also discussed *modifications to intervention delivery*: these could include changes to delivery agents (e.g. health practitioners vs. lay health workers), or format of delivery (e.g. face-to-face vs. media) [43, 46, 64]. Papers mentioned *context* as a potential target of modification, such as changes to locations or settings (e.g. community centre vs. church); however, we found little detail and guidance on how to implement contextual adaptations in practice [30, 33, 46, 54]. For example, Aarons et al. mention possible adaptations to the intervention’s inner (e.g. changes within the organisation where the intervention is delivered) and outer contexts (e.g. changes to funding and contracting to support implementation) [30]. Finally, *cultural adaptation* was often considered as a distinct type of adaptation broadly defined as changes to increase an intervention’s cultural relevance [33, 35, 42, 46, 60, 61, 64]. Beyond taking into account the broader socio-cultural, economic, and political factors, papers emphasised the importance of considering transverse cultural processes, exemplifying acculturative stress, phases of migration, developmental stages, availability of social support, and connections to the culture of origin [33]. These processes were highlighted to be of particular importance within the context of specific treatment adaptation and culturally sensitive delivery of psychotherapies.

##### Degrees of adaptation

In the context of cultural adaptation, papers drew on Resnicow et al. to distinguish between *surface* and *deep structure modifications* [65]. The former was reported as relating to harmonising intervention materials (e.g. handbooks as part of manualised interventions) to observable characteristics of the target population, such as using culturally appropriate messages, language, and product brands to improve outward appeal, acceptance, and face validity [35, 51, 56, 58, 61]. Deep structure adaptations, on the other hand, were commonly seen as aligning the intervention with core values, beliefs, norms, and worldviews to increase salience (e.g. incorporating collectivist values that emphasise interpersonal relationships in a health promotion intervention) [35, 43, 51, 56, 58, 61]. This distinction between surface and deep structure modifications was rather theoretical, and no guidance paper described a specific method for applying such a classification.

#### Category 4: Process of adaptation

**Fig. 2:**
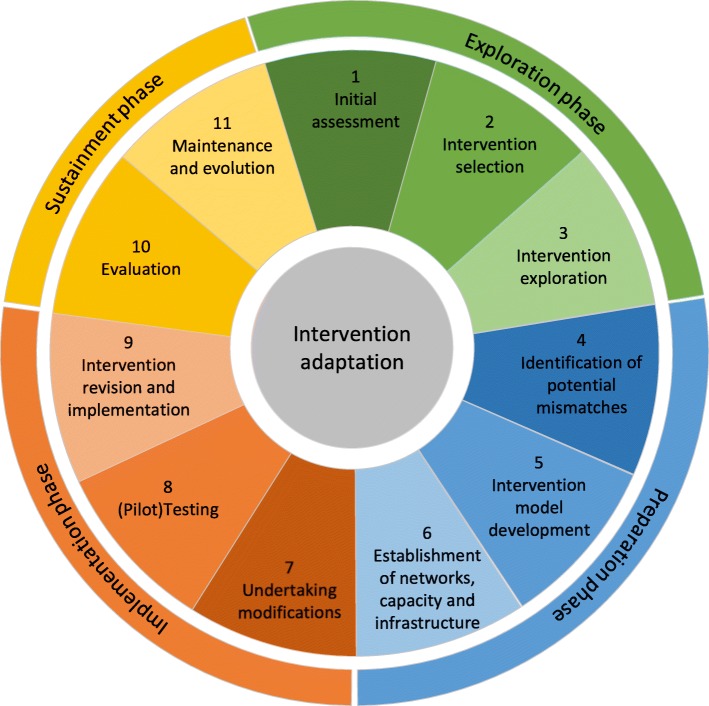
Overview of phases and steps in the process of adaptation

Most papers provided a stepwise approach to adaptation [29, 31, 32, 34, 36–38, 40, 41, 43, 45–53, 55–60, 62, 63, 66]. Based on the analysis of commonalities and differences in these approaches, we identified 11 unique steps for planning, conducting, and evaluating an adaptation and categorised them into four overarching phases of the EPIS implementation framework (see Fig. 2) [67]: *Exploration* (steps 1–3), *Preparation* (steps 4–6), *Implementation* (steps 7–9), and *Sustainment* (steps 10–11). Table 4 provides a short description of these steps (Additional file 5 presents the steps as described in each included paper and the frequency of reporting of each step across the papers).

**Table 4:** Summary of the key adaptation steps extracted from the guidance papers (*n* = 27)

	Step name	Step descriptions	Implemented by…
Exploration phase	1. Initial assessment	- Identify the need for a new intervention for the target population - Conduct a multilevel needs assessment of system, process, organisation, provider, and characteristics of the target population - Identify relevant contextual factors and community best practices	[29, 31, 32, 37, 41, 43, 45–49, 51–53, 55–57, 60, 63, 64, 66]
	2. Intervention selection	- Identify and review evidence-based interventions that address the public health problem of interest, risk behaviours, and environmental factors - Determine whether the intervention goals and outcomes are relevant to the target population - Determine whether the intervention content targets the population’s social and cultural values - Judge the fit of the intervention to the problem, organisational capacity, and target population - Select the best matching intervention	[32, 34, 38, 41, 45, 46, 48, 49, 52, 53, 55, 58, 60, 63, 64, 66]
	3. Intervention exploration	- Obtain the original intervention materials (e.g. statement of the goals, summary of the underlying theory of change, and/or the curriculum) - Identify the intervention’s core components and best-practice characteristics - Examine the theory base behind the intervention, identifying core mechanisms of change, moderators that may enhance or diminish outcomes, and any potential secondary pathways through which change might be achieved - Determine the interventions adaptability to the new target population and setting	[32, 34, 38, 47–51, 57–60, 62]
Preparation phase	4. Identification of potential mismatches	- Identify and categorise potential mismatches (e.g. among intervention goals, characteristics of the target population, implementation agency and/or community) - Identify potential implementation barriers - Identify potential barriers to participation - Assess fidelity/adaptation concerns for the particular implementation site, i.e. by determining what core components are especial to maintain to address fidelity	[29, 32, 34, 36, 38, 41, 45, 49, 52, 53, 56, 58, 62, 63]
	5. Intervention model development	- Define the extent of adaptation needed - Develop an overall logic model and timeline for adapting and implementing the intervention - Consider how components can accommodate population characteristics, delivery system, and community context - Explore potential ways to implement the adapted intervention and develop an implementation plan - Draft a user-friendly manual (i.e. “package”) of the intervention - Develop an overall implementation plan, including a strategy for achieving and measuring fidelity/adaptation balance for the selected intervention	[29, 34, 36, 41, 45–52, 57–59, 62, 63]
	6. Establishment of networks, capacity, and infrastructure	- Assess stakeholder input and potential collaborations and secure their meaningful involvement - Assess organisational, as well as implementers’ capacity to implement the intervention - Consult with the intervention developers, relevant organisational stakeholders, and the community to explore how they can help to shaped an implementation plan into a particular setting - Identify and recruit potential implementers, if possible with the same ethnic background as the target population (consider working with paraprofessionals or lay health workers) - Use community resources to increase intervention accessibility - Build community capacity for practical sustainability - Establish balance between community needs and scientific integrity by an iterative process among all relevant stakeholders involved in the adaptation process	[32, 36, 37, 40, 41, 45–47, 50, 52, 53, 56, 58–60, 62, 63, 66]
Implementation phase	7. Undertaking modifications	- Develop an adaptation plan - Consider adaptations that may be necessary to meet the needs of new target population, while making sure that core elements of the intervention’s programme theory are not altered - Consider possible local adaptations to improve cultural/context fit by taking into account potential language difference of cultural sub-groups - If applicable, develop a “mock-up” version of the adapted material, prepare design documents for adaptation, and draft user-friendly manuals of the intervention - Consider intervention training, including training of organisation staff - Adapt the relevant intervention components through collaborative efforts	[29, 32, 34, 37, 38, 40, 41, 43, 45–48, 52, 53, 56, 58, 60, 63, 66]
	8. (Pilot) testing	- Pilot test the adapted intervention components and procedures with representatives from the target group, get feedback and revise as necessary - Monitor the fidelity of intervention delivery	[31, 32, 37, 38, 40, 41, 45, 46, 48, 50, 52, 58, 60, 63]
	9. Intervention revision and implementation	- Refine adaptations based on results generated in previous steps - Synthesise stakeholder feedback and finalise the implementation plan - Implement the adapted intervention - Establish ongoing support, feedback and refinement	[29, 31, 37, 38, 40, 41, 45–47, 53, 60, 63]
Sustainment phase	10. Evaluation	- Decide how to evaluate and possibly incorporate feedback from diverse stakeholder groups and develop an evaluation plan that reflects the core mechanisms of change within the original programme theory, as well as adaptations made in intervention content to accommodate the new target population - Implement outcome evaluation - Provide routine, ongoing supervision (including quality assurance) - Assess acceptance of and participants’ engagement in the adapted intervention - Revise the intervention by adopting effective or dropping ineffective adaptations	[31, 32, 38, 41, 43, 45–47, 49, 50, 53, 56, 58, 63, 66]
	11. Maintenance and evolution	- Establish a wide-scale dissemination of the adapted intervention, given the intervention is successful and is embraced by the community - Develop training systems to widen the dissemination (e.g. train future implementers in the adapted version of the intervention) - Implement an ongoing re-assessment by circularising the process and outcome research results and the lessons learned in the transportation of the evidence-based intervention	[29, 41, 53, 66]

Before implementing an adaptation, many papers highlighted the value of an exploration phase, including an initial assessment (step 1) to identify the needs of the target population, the system, the organisational capacity, and thereby the need for a new intervention. After this, it is important to select the appropriate intervention for adaptation (step 2) involving identification of relevant evidence-informed interventions, judgment of their fit with the new context, and selection of the best match. The selected intervention is then examined (step 3) for its components and theory to determine its adaptability to the new context. Following these, there are several steps to prepare for the adaptation, including identification of potential mismatches (step 4), development of an intervention model (step 5), and establishment of important networks and capacity (step 6). The next phases are concerned with the actual undertaking of the adaptations, including development of the adaptation plan (step 7), pilot testing of the proposed adaptations (step 8), and revisions and implementation of the adapted intervention (step 9). Finally, the adapted intervention is evaluated (step 10) both for important outcomes and for the establishment of routine and ongoing supervision and monitoring. The last step (step 11) involves activities to disseminate the adapted intervention and sustain it through training systems and ongoing re-assessments.

Paper authors described how these steps did not necessarily follow a linear process. In line with best practice in intervention development [68, 69], individual steps within the four phases were often described to take place in parallel or had a different order across the papers. Furthermore, there were differences in phase attribution. For example, some papers outlined establishment of relevant networks (preparation phase, step 6) as a sub-step in the initial assessment step (exploration phase) [36, 37, 45–47, 56, 58]. In contrast, some papers prioritised an in-depth needs assessment at the beginning of the adaptation process (exploration phase) [32, 41, 62, 63].

#### Category 5: Stakeholder involvement

Papers recommended a range of stakeholders to involve in adaptation. While different papers emphasised different stakeholders, we categorised the most commonly reported stakeholders into five main groups: *(i) local community leaders, partners,* and *implementers* [29, 31, 32, 34, 36–41, 45–47, 49, 50, 52–56, 58–62]; *(ii) representatives of the target population* [31, 32, 34, 36–41, 44–47, 49, 52, 53, 55, 58, 60, 61, 63]; *(iii) intervention developers* and *topic experts* [29, 31, 36, 45, 48, 52, 54, 58, 62, 63]; *(iv) researchers* [29, 36, 43, 46–48, 55, 59, 60]; and *(v) practitioners* and *policy-makers* [29, 31, 32, 34, 36–41, 43–56, 58–63, 66]. Involvement of policy-makers in intervention adaptation was mentioned in only two papers [58, 66], perhaps reflecting the predominant focus of papers on micro- and meso-level interventions (see Table 1).

Papers described different ways to involve these groups of stakeholders across different steps of adaptation. For example, needs assessment through formative research and pilot testing was commonly proposed to engage and learn from the local community partners and implementers during the exploration phase of adaptation [29, 31, 36–41, 45–47, 50, 52–54, 58, 60, 62, 63, 66]. As part of preparation and implementation phases, meetings and consultations were suggested with intervention developers and topic experts to guide the adaptation process and monitor fidelity [29, 45, 48, 52, 62, 63]. Many papers recommended following the CBPR approach to engage with and seek input from community partners and representatives of the target population [31, 34, 36–38, 40, 41, 45–47, 49, 52, 53, 56, 59–61, 66]. For example, focus groups or elicitation interviews were often discussed as a way to assess local capacity, resources, and preferences. More innovative methods, such as theatre testing, which involve representatives of the target population responding to a demonstration of an adapted intervention were also suggested [52].

A few papers highlighted the value of forming specific stakeholder committees to lead the entire process of adaptation [29, 34, 36, 41, 60, 66]. A range of stakeholders from those listed above were recommended for inclusion in these committees depending on the topic and level of the intervention. These committees were differently referred to in the papers as a Community Advisory Board [60], a Community Working Group [41], and an Implementation Research Team [29].

#### Category 6: Evaluating adapted interventions

Papers often highlighted the need for additional testing of effects of an adapted intervention in a new context, however did not offer an explicit rationale for such an evaluation or guidance for choosing and prioritising among different evaluation approaches and methods. *Outcome evaluation* was the most frequently reported approach [30, 34, 35, 38, 41, 46, 47, 49, 50, 52] with a range of specific methods discussed, including different types of randomised and non-randomised study designs. *Process evaluation* [30, 34, 38, 41, 45–47, 49, 66], *piloting* [31, 35, 45, 52, 58, 63], and *fidelity monitoring* [29, 41, 50, 54, 57, 58] were also commonly mentioned. These approaches were described separately, most frequently as part of the distinct steps in the adaptation process. Table 5 provides further details on a range of approaches for re-evaluation, including the rationale and specific methods for each approach.

**Table 5:** Approaches for re-evaluating an adapted intervention

Approach	Rationale	Specific methods
Formative evaluation^a^ [47]	- Identify factors affecting intervention design, success, and sustainability (e.g. community resources, population characteristics) - Inform adaptation	- Formative research - Input from stakeholders prior to or during adaptation
Pilot testing [31, 35, 45, 52, 58, 63]	- “Dress rehearsal” to inform revisions - Identify difficulties with implementation and sources of non-fit - Identify anticipated immediate outcomes - Provide adaptation data for other researchers - Assess satisfaction with and acceptability of the intervention	- Process-oriented qualitative data using in-depth interviews and focus groups with key stakeholders - Short-term/small-scale trials - Assessment of engagement constructs and making comparisons with similar data from published studies
Process evaluation [30, 34, 38, 41, 45–47, 49, 66]	- Identify context-specific factors affecting intervention effectiveness in a new context (i.e. context-specific mediators and moderators) - Document implementation and adaptation processes (e.g. activities implemented, how and with whom) - Identify factors affecting intervention implementation - Determine the intervention reach - Determine acceptability of and satisfaction with the intervention - Identify suggested improvements - Determine the usefulness of the adapted interventions - Document successes and barriers to inform future adaptations	- Self-reported measures - Qualitative methods (e.g. interviews, notes, site visits by intervention developers, the adaptation team, and send case videotapes to intervention developers) - Quantitative methods (e.g. weekly session ratings) - Mixed-methods approaches
Fidelity assessment/monitoring [29, 41, 50, 54, 57, 58]	- Ensure true replication of the intervention by assessing the degree of adherence to delivering the intervention (e.g. whether the core elements have been successfully implemented) - Assess the adapter’s competence in delivering the intervention - Ensure intervention quality maintenance	- A phased approach using assessment of the process documentation forms, discussion with a group of developers, refinement of the assessment through discussions with implementers - Fidelity monitoring tool/checklist (e.g. by using direct observations and ratings) - Qualitative interviews - Assessment of notes, client reports
Large-scale implementation evaluation [30]	- Assess impact on the mediating variables - Make inferences about the changes of the distal outcomes	- Assessment of proxy or indirect measures of the key RE-AIM^b^ components
Core component mediational analysis (also termed as mechanisms evaluation) [35, 39, 58, 66]	- Determine which components of an intervention most influence intervention effectiveness - Inform the need for further adaptations - Inform the need for a larger scale dissemination trial	- Experimental dismantling designs (e.g. a three-arm effectiveness trial using (1) a minimally adapted version of the intervention, (2) a fully adapted version of the intervention, and (3) a treatment as usual.
Outcome evaluation (also termed as a summative evaluation) [30, 34, 35, 38, 41, 46, 47, 49, 50, 52]	- Assess the effectiveness of interventions in new contexts/with new populations - Assure achievement of expected outcomes (proxy, short-term, as well as distal outcomes) - Inform future implementation and dissemination efforts - Gather evidence on vulnerable populations underrepresented in clinical/efficacy research	- Use of a control condition, random assignment - Type 2 hybrid trial testing both effectiveness and implementation (baseline survey, process measures, and at least 3-month post-intervention assessment) - Small-scale or cluster randomised controlled trials (RCTs) - Alternatives to RCTs that are more context-specific^c^ (e.g. propensity score matching, interrupted times series) - Collection of data on community-level outcomes (e.g. social networks, resources, and community capacity levels) - Pretest/posttest designs and comparison with literature
Comparison evaluation [35]	- Assess the superiority of the adapted intervention over standard interventions	- A large RCT comparing the adapted interventions with a standard intervention
Cost-benefit assessment [41, 66]	- Assess whether the extra costs of intervention adaptation are justified - Support the case for the intervention adaptation to stakeholders	- Cost-benefit analysis

^a^Procedures conducted while the intervention is still forming (i.e. in progress)

^b^The RE-AIM framework components include Reach, Effectiveness, Adoption, Implementation, and Maintenance

^c^RCTs may not be feasible in community settings because researchers have less control over intervention delivery, use of usual-care control groups may be unethical, contamination might be an issues, and resistance to randomisation may be heightened in racial/ethnic minority communities

We found only one paper proposing a strategy to determine the level of empirical evidence required for an adapted intervention to retain its evidence-informed standard in a new context [30]. Aarons et al. make a conceptual argument for the possibility to “borrow strength” from evidence in the original effectiveness study to allow for a more limited evaluation when scaling-out interventions to new populations and/or using new delivery systems [30]. This would include, for instance, using implementation evaluation rather than a new effectiveness study when a strong case can be made for similar mechanisms between original and new contexts. The authors’ statements were largely theoretical, and they argue that these require further empirical testing.

## Discussion

### Main findings in the context of other research

This study is the first systematic review of guidance on adapting complex population health interventions to new contexts. The review explores the content of 35 papers and sheds light on contested issues by providing a thorough synthesis of key concepts frequently used in adaptation research (see Table 3) and a comprehensive overview of adaptation processes (see Fig. 2 and Table 4). The review explicates the overall aims of adaptation, which are largely framed as enhancing cultural relevance and the sense of local ownership of the intervention with explicit commitment to the principles of CBPR and Roger’s diffusion of innovations theory (see Table 1) [70]. Both perspectives highlight the sense of local ownership as an important driver of intervention acceptability and adoption [71, 72].

Our findings are largely consistent with those of the previous scoping study of adaptation frameworks [15]. As in the previous scoping study, we identified 11 common steps of adaptation. However, our review further extends the previous work through a systematic approach and a broadened scope, including additional insights from papers on cultural adaptation and a range of topic areas beyond public health. In line with the previous scoping study [15], our review also finds relatively widespread agreement in key concepts and the process of adaptation.

### Strengths and limitations of this systematic review

This review consolidates existing guidance on adaptation following systematic searches in databases complemented by expert consultations to help locate additional sources. Its key strengths lie in the thorough exploration and synthesis of the content of the existing guidance following best practices in systematic reviewing, including a broad search strategy in terms of databases and search terms, double screening and data extraction, pilot testing of the extraction form, and an evidence synthesis strategy combining deductive and inductive approaches.

There were some limitations. First, while we aimed to include papers in a range of languages, searches were conducted in English, potentially missing relevant non-English papers. Second, while we included a range of terms related to adaptation in the search strategy (e.g. replication, transfer), we might have missed terms used synonymously by other researchers. This particularly relates to guidance papers on macro-level interventions, which were underrepresented in our review, as adaptation may be framed and conceptualised differently for these broader types of interventions (e.g. policy changes rather than adaptations). Third, we had to use a strict definition of guidance for practical considerations and had rounds of discussions within the author team to determine eligibility. As shown in Table 1, we included only papers which explicitly provide recommendations for practice. Many papers, which did not meet this definition, but provided important discussions on intervention adaptation, were left out, such as the classification of adaptations by Stirman et al. [16] and works on cultural adaptation by Castro et al. [17] and Resnicow et al. [65]. It should, however, be noted that the included papers often referred to these conceptual resources to support their definitions and recommendations. We can thus argue that much of the thinking in these resources has shaped the guidance papers included in our review. Finally, we did not assess the utility of the guidance papers from a user’s perspective and used data as reported. Our quality appraisal, for example, may have missed important information that was not included in the papers. However, we used pre-defined criteria for quality appraisal to enhance the rigour and transparency of our approach.

### Limitations of existing guidance and recommendations for future research and guidance

While our review found large agreement with respect to terminology, reasons, types, and processes of adaptation, the papers included in our review have predominantly been developed and applied in the USA. The key governmental agencies supporting the work on adaptation frameworks have been those responsible for child health, the control of infectious diseases and substance abuse, which explains the predominant focus on topics, such as sexually transmitted infections (STIs), HIV prevention, and parenting. This questions whether the current guidance adequately reflects intervention adaptation for a broader set of topic areas and across a broader range of countries, particularly when adapting interventions to low- and middle-income settings with varying levels of resources and systems of provision.

Our review also identified important gaps in scope, conceptualisation, and operationalisation in the existing guidance. First, as noted above, the available guidance has a predominant focus on micro-level (behavioural) interventions and their transfer to specific sub-groups. While important insights for adaptation can be gleaned from this body of work, the applicability of the suggested procedures for transferring broader macro-level interventions across countries and continents might be questioned. This includes, for example, potential challenges associated with using CBPR principles and procedures (as widely discussed in the current papers) and engaging with policy-makers at national-level institutions and decision-making. While papers published later in the 2010s were more inclusive of meso-level interventions (see Table 1), they were few in our review. This suggests a need for additional research on scaling-out and adaptation of meso- and macro-level interventions (e.g. policy interventions), including literature reviews using tailored searches to identify studies, that may use a different framing of and terms for adaptation.

Our findings also suggest lack of theorisation in terms of intervention mechanisms and broader systems thinking in the existing guidance [3, 4]. It is increasingly recognised that interventions represent events in complex systems and that their effects are a result of interactions between context, implementation, and intervention design itself [73, 74]. At present, this perspective is not adequately reflected and operationalised in the existing guidance. Interventions are exclusively seen as relatively fixed and bounded entities consisting of a set of distinct components (core or discretionary), the presence, absence, or combinations of which are seen as responsible for the observed outcomes without explicitly linking them with intervention mechanisms. Furthermore, while there is a common emphasis on the need to distinguish between intervention core and discretionary components, we did not find guidance on how to identify the core components that need to be maintained during adaptation. If this was intended to be accomplished through engagement with the developers of the original intervention, it was not transparently discussed in the papers as a key purpose of such engagement. It is important that future guidance more transparently describes the processes of intervention exploration (see Fig. 2), including the specific roles of key stakeholders and management of potential conflicts that may arise from their involvement. In general, transparent reporting of an interventions’ theory and how it may be implemented in practice (i.e. theory-based strategies) will also be informative for adaptation research [75, 76]. While a lot of work has been done on articulating intervention theories in the literature on the development and evaluation of interventions [68, 77], it does not seem to have adequately translated into adaptation research, and future work should aim to fill this gap.

Key concepts, such as adaptation and fidelity (see Table 3), are also largely conceptualised in relation to intervention form, that is, specific design features rather than intervention mechanisms and functions. While intervention components can be linked to specific mechanisms, as highlighted by Hawe et al. [18], it is arguably not the design components that need to be standardised, but rather the aspects of intervention mechanisms that these components are aiming to facilitate. Similarly, there is a lack of critical engagement with different types of context. Where discussed, it is primarily seen as a factor facilitating or impeding implementation rather than as an inherent and active element in the construction of intervention effects. No paper directly guides the user to critically examine the underlying mechanisms and the possible contextual interactions that could matter in the replicability of the effects [78, 79]. While many papers highlight the potential mismatches in the contextual characteristics between the original and the new contexts as important pre-requisites for adaptation, they do not discuss the implications of these mismatches for intervention effects in a new context. This is a particularly important issue to tackle in light of the evidence suggesting no added benefits associated with extensively adapted interventions [5] alongside the evidence in favour of these adaptations [7, 80]. Future guidance needs to reflect more critically on intervention mechanisms and contextual interactions to inform decisions on the need and the extent of adaptation that might be warranted. There is a growing body of literature on how to design and implement interventions in a context-sensitive manner [12, 74, 77, 81]. This involves, for example, delineation of important contextual characteristics, such as epidemiological, socio-cultural, socio-economic, ethical, legal, and political factors [81] and theorisation and testing of how intervention effects may be contingent upon these factors.

Another key gap relates to the appropriate evaluation of adapted interventions. While different approaches to and methods of evaluation are described, ranging from feasibility studies to full-scale randomised evaluation studies, no guidance is given on how to choose among these methods. Full-scale evaluation of an adapted intervention can be costly and resource-intensive, and further research should empirically test the conceptual arguments set forth by Aarons et al. on the possibility of an adapted intervention to “borrow strength” as a potentially efficient approach [30]. In a similar vein, our review identified a range of stakeholders to be consulted during the adaptation process. While stakeholder involvement is widely viewed as positive in achieving greater acceptability and fit of the intervention, it might be associated with additional financial and human resource costs [82]. Further research and testing on which stakeholders should be prioritised during which phase of the adaptation process and on the optimal types and levels of involvement is warranted to provide efficient solutions.

Finally, as highlighted previously [14], further guidance also needs to be established on how best to document and report intervention adaptation. Although our review aimed to extract data on intervention reporting, we did not find any guidance providing recommendations for adaptation reporting. Recently, Stirman and colleagues published the updated Framework for Reporting of Adaptations and Modifications to Evidence-based interventions (FRAME) approach [83]. While the framework largely focuses on documentation of adaptations during the implementation process, it includes a range of items potentially applicable for different contexts of adaptation (e.g. planned and unplanned adaptations). Further application and testing of this framework would be warranted in the context of planned intervention adaptations to new contexts.

### How to use this systematic review?

We see three broad uses of our review. First, it can serve as a catalogue of existing guidance papers on adaptation and therefore aid researchers and practitioners in easily locating relevant resources to consult for their context of work. Second, by synthesising existing recommendations and delineating important gaps, this review contributes in setting the research agenda for future methodological work on adaptation where further innovation would be required. Specifically, the review findings inform the next steps of the ADAPT Study. The synthesis of the key concepts (see Table 3), and highlighting areas of clarity and uncertainty in conceptual thinking, will inform the planned overarching guidance. It is important to further problematise the review findings in light of the key gaps and seek further input and agreement around contested issues, such as conceptualisation of fidelity in relation to intervention form vs. intervention function, differentiation between intervention core vs. discretionary components, and the language used around these concepts and procedures. Some of our review findings, specifically the phases and steps of adaptation (see Table 4 and Fig. 2), provide clear first drafts for structuring the ultimate guidance and will serve as the starting point for the next stages of the study. Issues such as whether the number of steps in the process of adaptation as identified in this review is practical and how the steps may be further revised and optimised will be explored in the next stages of the ADAPT Study, which include a scoping review of cases of intervention adaptation followed by qualitative interviews with a range of stakeholders, such as researchers, editors, and funders to examine adaptation practices and compare those with the existing guidance. Subsequently, an international Delphi panel will be convened, where key considerations and issues from previous stages, including this review, will be examined and refined through several rounds of revisions and feedback [11]. Finally, our review findings can provide those adapting interventions to new contexts with interim tools to consult until the ADAPT Study guidance is available. It should, however, be noted that we do not intend for our review findings to be used as a source of expert advice on adaptation, but rather that it should be considered reflectively as a descriptive synthesis of existing concepts and recommendations.

## Conclusion

This systematic review synthesises currently available guidance on adapting interventions to new contexts. It can be used as a resource for researchers, policy-makers, and practitioners working to adapt interventions to new contexts. By highlighting important gaps in the field, the findings also serve to inform future methodological work and guidance development on adaptation. The findings will be used to inform the ADAPT Study guidance on adapting population health interventions to new contexts.

## Supplementary information

**Additional file 1.** Systematic review PRISMA checklist. This additional file provides the completed PRISMA checklist for the systematic review

**Additional file 2.** Search strategy. This additional file provides the full search strategy used in the review

**Additional file 3.** Data extraction template. This additional file provides the template used for data extraction, including details on the criteria used to assess quality of the included papers

**Additional file 4.** Appraisal of the included guidance papers. This additional files provides the ratings of the included studies against the pre-defined quality appraisal criteria

**Additional file 5.** Adaptation steps and phases. This additional file provides adaptation steps as originally reported in the included studies, as well as and the frequency of reporting of each step across the papers)

## Data Availability

Further data and materials are included in the additional files.

## Abbreviations

CBPR: Community-based participatory research
MRC: Medical Research Council
