# Supplementary material for: Adapting evidence-informed complex population health interventions for new contexts: a systematic review of guidance
Source: Implement Sci. 2019 Dec 17;14:105. doi: 10.1186/s13012-019-0956-5 (PMC6918624; doi:10.1186/s13012-019-0956-5)
Supplement: Supplementary file 2 — Additional file 2. Search strategy. This additional file provides the full search strategy used in the review [file 13012_2019_956_MOESM2_ESM.docx]

# Review search strategy

**All searches were conducted on 12 Oct 2018**

**Ovid MEDLINE(R) and Epub Ahead of Print, In-Process & Other Non-Indexed Citations, Daily and Versions(R)** 1946 to October 08, 2018

|  | *evidence-based practice/ or *evidence-based medicine/ | 28,164 |
| --- | --- | --- |
|  | exp *Health Promotion/ | 46,218 |
|  | (evidence based or evidence-informed or EBP? or EBI? or EBT? or EBM?).ti,ab,kf. | 110,669 |
|  | (health promotion or population impact).ti,ab,kf. | 29,201 |
|  | ((effective or efficacious) adj3 (program? or programme? or intervention? or treatment?)).ti,ab,kf. | 171,296 |
|  | or/1-5 | 349,882 |
|  | Program Evaluation/ | 57,594 |
|  | (method* or guideline? or guidance or guide or framework? or system? or tool? or process*2 or approach*2 or best practice? or good practice? or model? or recommendation? or step? or challenge? or principle? or standard?).ti,kf. | 2,384,751 |
|  | (method* or guideline? or guidance or guide or framework? or system? or tool? or process*2 or approach*2 or best practice? or good practice? or model? or recommendation? or step? or challenge? or principle? or standard?).ab. /freq=3 | 2,939,077 |
|  | or/7-9 | 4,460,195 |
|  | 6 and 10 | 114,549 |
|  | Program Development/ | 27,041 |
|  | Cultural Diversity/ | 10,702 |
|  | (implement* or develop* or prevent* or disseminat*).ti,kf. | 984,639 |
|  | (implement* or develop* or prevent* or disseminat*).ab. /freq=3 | 637,885 |
|  | ((new or diverse or different or other) adj (context? or population? or communit* or culture? or setting?)).ti,ab,kf. | 59,259 |
|  | ((ethnocultural or ethno-cultural) adj (group? or context? or population? or communit* or setting?)).ti,ab,kf. | 171 |
|  | or/12-17 | 1,477,715 |
|  | 11 and 18 | 31,662 |
|  | Diffusion of Innovation/ | 16,637 |
|  | ((intervention? or program? or programme? or policy or policies or treatment? or guideline? or psychotherap*3) adj5 (adapt* or modif* or transfer* or transport* or translat* or replicat* or tailor*)).ti,kf. | 10,675 |
|  | ((intervention? or program? or programme? or policy or policies or treatment? or guideline? or psychotherap*3) adj5 (adapt* or modif* or transfer* or transport* or translat* or replicat* or tailor*)).ab. /freq=2 | 9,577 |
|  | (intervention fidelity or implementation fidelity or external validity or scaling-out or scale-out or transferability).ti,ab,kf. | 7,580 |
|  | ((cultural* or transcultural* or crosscultural*) adj (adapt* or modif* or relevan* or competen* or tailor* or appropriat* or sensitiv*)).ti,ab,kf. | 18,325 |
|  | or/20-24 | 60,148 |
|  | 19 and 25 | 2,315 |
|  | (population health/ or complex intervention?.ti,ab,kf.) and (adaptation or spread or modification or dissemination or translation).ti,ab,kf. | 95 |
|  | 26 or 27 | 2,399 |
|  | Limit 29 to yr=“2000-Current” | 2,315 |

**Ovid Embase (1974 to 2018 October 11)**

|  | *evidence-based practice/ or *evidence-based medicine/ | 27,152 |
| --- | --- | --- |
|  | exp *Health Promotion/ | 35,097 |
|  | (evidence based or evidence-informed or EBP? or EBI? or EBT? or EBM?).ti,ab. | 141,182 |
|  | (health promotion or population impact).ti,ab. | 30,945 |
|  | ((effective or efficacious) adj3 (program? or programme? or intervention? or treatment?)).ti,ab. | 242,733 |
|  | or/1-5 | 439,815 |
|  | Program Evaluation/ | 11,263 |
|  | (method* or guideline? or guidance or guide or framework? or system? or tool? or process*2 or approach*2 or best practice? or good practice? or model? or recommendation? or step? or challenge? or principle? or standard?).ti. | 2,407,643 |
|  | (method* or guideline? or guidance or guide or framework? or system? or tool? or process*2 or approach*2 or best practice? or good practice? or model? or recommendation? or step? or challenge? or principle? or standard?).ab. /freq=3 | 3,909,833 |
|  | or/7-9 | 5,269,118 |
|  | 6 and 10 | 140,507 |
|  | Program Development/ | 22,383 |
|  | Cultural Diversity/ | 978 |
|  | (implement* or develop* or prevent* or disseminat*).ti. | 994,156 |
|  | (implement* or develop* or prevent* or disseminat*).ab. /freq=3 | 861,696 |
|  | ((new or diverse or different or other) adj (context? or population? or communit* or culture? or setting?)).ti,ab. | 73,495 |
|  | ((ethnocultural or ethno-cultural) adj (group? or context? or population? or communit* or setting?)).ti,ab. | 199 |
|  | or/12-17 | 1,672,275 |
|  | 11 and 18 | 35,440 |
|  | Diffusion of Innovation/ | 12,267 |
|  | ((intervention? or program? or programme? or policy or policies or treatment? or guideline? or psychotherapy*3) adj5 (adapt* or modif* or transfer* or transport* or translat* or replicat* or tailor*)).ti. | 12,564 |
|  | ((intervention? or program? or programme? or policy or policies or treatment? or guideline? or psychotherp*3) adj5 (adapt* or modif* or transfer* or transport* or translat* or replicat* or tailor*)).ab. /freq=2 | 13,175 |
|  | (intervention fidelity or implementation fidelity or external validity or scaling-out or scale-out or transferability).ti,ab. | 8,562 |
|  | ((cultural* or transcultural* or crosscultural*) adj (adapt* or modif* or relevan* or competen* or tailor* or appropriat* or sensitiv*)).ti,ab. | 21,160 |
|  | or/20-24 | 64,766 |
|  | 19 and 25 | 1,902 |
|  | (population health/ or complex intervention?.ti,ab.) and (adaptation or spread or modification).ti,ab. | 115 |
|  | 26 or 27 | 2,011 |
|  | Limit 29 to yr=“2000-Current” | 1,963 |

**EBSCOhost PsycINFO(R)**

|  | DE “evidence-based practice” | 1,963 |
| --- | --- | --- |
|  | DE “health promotion” | 26,509 |
|  | TX (“evidence-based” or “evidence-informed” or EBP$ or EBI$ or EBT$) | 36,958 |
|  | TX (effective or efficacious or preventive) n3 (program$ or programme$ or intervention$ or treatment$) | 42,897 |
|  | or/1-4 | 100,595 |
|  | DE “program evaluation” | 18,549 |
|  | TI (method* or guideline$ or guidance$ or guide or framework$ or system$ or tool$ or process$$ or approach$$ or “best-practice$” or “good-practice$” or model$ or recommendation$ or standard$) | 263,998 |
|  | AB (method* or guideline$ or guidance$ or guide or framework$ or system$ or tool$ or process$$ or approach$$ or “best-practice$” or “good-practice$” or model$ or recommendation$) | 1,499,769 |
|  | or/6-8 | 1,542,469 |
|  | 5 and 9 | 71,136 |
|  | DE “program development” | 7,991 |
|  | DE “cultural sensitivity” | 3,680 |
|  | TI ((implement* or develop* or prevent* or disseminat*) | 143,933 |
|  | AB (implement* or develop* or prevent* or disseminat*) | 780,141 |
|  | TI ((new or diverse or different or other) n1 (context$ or population$ or communit* or culture$ or setting$)) | 1,870 |
|  | AB ((new or diverse or different or other) n1 (context$ or population$ or communit* or culture$ or setting$)) | 34,632 |
|  | AB ((ethnocultural or ethno-cultural) n1 (group$ or context$ or population$ or communit* or setting$)) | 166 |
|  | or/11-17 | 829,114 |
|  | 10 and 18 | 40,317 |
|  | TI ((intervention$ or program$ or programme$ or policy or policies or treatment$ or guideline$ or psychotherap*) n3 (adapt* or modif* or transfer* or transport* or translat* or replicat* or tailor*)) | 2,228 |
|  | AB ((intervention$ or program$ or programme$ or policy or policies or treatment$ or guideline$) n3 (adapt* or modif* or transfer* or transport* or translat* or replicat*)) | 13,022 |
|  | TI ((cultural* or transcultural* or crosscultural*) n1 (adapt* or modif* or relevan* or competen* or tailor* or appropriat* or sensitiv*)) | 2,776 |
|  | AB ((cultural* or transcultural* or crosscultural*) n1 (adapt* or modif* or relevan* or competen* or tailor* or appropriat* or sensitiv*)) | 12,400 |
|  | AB (intervention fidelity or implementation fidelity or external validity scaling-out or scale-out or transferability) | 1,751 |
|  | or/20-24 | 28,424 |
|  | 19 and 25 | 2,818 |
|  | Limit 26 to yr=“2000-Current” | 2,732 |

**ProQuest Social Sciences**

**Applied Social Sciences Index & Abstracts (ASSIA) (1987 – current)**

**Dissertations and Theses Global: The Humanities and Social Sciences Collection (1637 – current)**

|  | ALL(evidence-based or evidence-informed or EBI? or EBP?) | 22,765 |
| --- | --- | --- |
|  | AB,TI ((effective or efficacious or preventive) n/3 (program? or programme? or intervention? or treatment?)) | 6,561 |
|  | or/1-2 | 29,281 |
|  | AB,TI(method* or guideline? or guidance or guide or framework? or system? or tool? or process?? or approach?? or best-practice? or good-practice? or model? or recommendation? or standard?) | 1,511,787 |
|  | 3 and 4 | 20,305 |
|  | AB,TI(implement* or develop* or prevent* or disseminat*) | 971,431 |
|  | AB,TI((new or diverse or different or other) n/1 (context? or culture? or setting? or communit*)) | 36,244 |
|  | or/6-7 | 990,122 |
|  | 5 and 8 | 12,632 |
|  | AB,TI((intervention? or program? or programme? or policy or policies or treatment? or guideline?) n/3 (adapt* or modif* or transfer* or transport* or replicat* or tailor*)) | 21,553 |
|  | AB,TI((cultural* or transcultural* or crosscultural*) n/1 (adapt* or modif* or relevan* or competen* or tailor* or appropriat* or sensitiv*)) | 15,481 |
|  | AB,TI (intervention fidelity or implementation fidelity or external validity scaling-out or scale-out or transferability) | 7,993 |
|  | or/10-12 | 44,210 |
|  | 9 and 13 | 1,301 |
|  | Limit 14 to yr=“2000-Current” | 1,128 |

**Web of Science Core Collection: Citation Indexes**

**Social Sciences Citation Index (SSCI) (1990 – present)**

**Conference Proceedings Citation Index – Social Science & Humanities (CPCI-SSH) (1990 – present)**

|  | TS=(“evidence-based” or “evidence-informed”) | 40,239 |
| --- | --- | --- |
|  | TS=((effective or efficacious) near/3 (program$ or programme$ or intervention$ or treatment$)) | 38,890 |
|  | or/1-2 | 76,727 |
|  | TS=(method* or guideline$ or guidance or guide or framework$ or system$ or tool$ or process or processes or approach or approaches or “best-practice$” or “good-practice$” or model$ or recommendation$) | 2,629,178 |
|  | 3 and 4 | 59,618 |
|  | TS=(implement* or develop* or prevent* or disseminat*) | 1,404,540 |
|  | TS=("new context$" or "new population$" or "new community" or "new communities" or "new culture$" or "new setting$" or "diverse population$" or "diverse context$" or "ethnocultural population$" or "ethnocultural group$" or "different context$" or "different population$" or "different setting$") | 14,951 |
|  | or/6-7 | 1,412,754 |
|  | 5 and 8 | 36,966 |
|  | TS=((intervention$ or program$ or programme$ or policy or policies or treatment$ or guideline$) near/3 (adapt* or modif* or transfer* or transport* or replicat*)) | 22,272 |
|  | TS=((cultural* or transcultural* or crosscultural*) near/1 (adapt* or modif* or relevan* or competen* or tailor* or appropriat* or sensitiv*)) | 18,496 |
|  | TS=(“intervention fidelity” or “implementation fidelity” or “external validity” or “scaling-out” or “scale-out” or transferability) | 5,363 |
|  | or/10-12 | 45,108 |
|  | 9 and 13 | 2,437 |
|  | Limit 14 to yr=“2000-Current” | 2,366 |
