# Supplementary material for: Adapting evidence-informed complex population health interventions for new contexts: a systematic review of guidance
Source: Implement Sci. 2019 Dec 17;14:105. doi: 10.1186/s13012-019-0956-5 (PMC6918624; doi:10.1186/s13012-019-0956-5)
Supplement: Supplementary file 3 — Additional file 3. Data extraction template. This additional file provides the template used for data extraction, including details on the criteria used to assess quality of the included papers [file 13012_2019_956_MOESM3_ESM.docx]

**Additional File 2.**

**Table 1.** Data extraction template

| **Descriptive information** | | | | |
| --- | --- | --- | --- | --- |
| Extractor initials: |  | | | |
| Date extracted: |  | | | |
| Author(s) of the document: |  | | | |
| Publication year: |  | | | |
| Publication source: |  | | | |
| Document title: |  | | | |
| Document focus: |  | | | |
| **Rationale and circumstances requiring an adaptation** | | | | |
| Rationale and aims of adaptation | Rationale for and aims of an adaptation described by the authors of the guidance: why should an intervention be adapted? | | | |
| Pre-requisites for adaptation | Pre-requisites for undertaking an adaptation: when to do an adaptation? Work needed before the appropriateness for such an adaptation can be established. Evidence needed to justify conducting an adaptation (e.g., contextual incongruence). | | | |
| Generalisability (how to assess generalisability to other contexts?) | How to assess generalisability/applicability of the interventions’ evidence to other contexts? | | | |
| **Key concepts/nomenclature and their definitions related to intervention adaptation**  **(e.g., adaptation, scale-out, fidelity, core elements)** | | | | |
| Concept | Definition/description | | | |
| **Types and the extent of adaptation: what should be adapted and how much?**  **(e.g., adaptation to intervention, implementation, or context, intervention form or function)** | | | | |
| Type | Definition/description | | Example(s) | Extractor’s comments |
| The extent of adaptation | Definition/description | | Example(s) | Extractor’s comments |
| **Process of adaptation: how to do an adaptation**  **(e.g., phases of adaptation)** | | | | |
| Overview of the process | Definition/description | | | |
| Process/phases | Definition/description | | Example(s) | Extractor’s comments |
| Stakeholder engagement | Definition/description | | | |
| **Methodological approaches for re-evaluating adapted interventions: why and how to re-evaluation the adapted intervention?** | | | | |
| Rationale for re-evaluation or no re-evaluation | Rationale for intervention re-evaluation described by the authors of the guidance. Should adapted interventions be re-evaluated and to what extent? | | | |
| Feasibility/piloting | Should adapted interventions be tested for feasibility/piloting and why? | | | |
| Full-scale evaluation | Should adapted interventions be evaluated in full-scale and why (e.g., assessing effectiveness and change processes)? | | | |
| Implementation | Can adapted interventions be implemented without evaluation and why? | | | |
| Specified approach to re-evaluation | Definition/description | | | Extractor’s comments |
| **Methodological approaches to assessing the process of adaptation: why and how to evaluation the adaptation process?** | | | | |
| Rationale for assessing the process of adaptation | Rationale for assessing the process of intervention adaptation described by the authors of the guidance. Should there be an assessment of the adaptation process and to what extent? | | | |
| Specified approach to assessing the process of adaptation | Definition/description | | | Extractor’s comments |
| **Reporting**  **(e.g., any recommendations or criteria on what/how to report intervention adaptation)** | | | | |
| Criterion/recommendation | Definition/description | | | Extractor’s comments |
| **Diagrams from the documents**  **(e.g., figures depicting the adaptation types or the process)** | | | | |
| Diagram name | Diagram link to the text (i.e., whether it relates to the types, processes or methodological approaches) | | | Diagram (copy) |
| **Other emergent issues** | | | | |
| Issue | | Definition/description | | Extractor’s comments |

**Table 2.** Criteria for assessing characteristics of guidance papers

| **Criterion** | **Rating** | **Coder’s comments** |
| --- | --- | --- |
| Practicality | | |
| Understandability and clarity  Guiding question:   - *Are the key constructs/procedures clearly specified?* - *Are the relationships between the key constructs and/or the chain of the procedures clearly specified?* | –/+/++ |  |
| Ease of use and operationalisability  Guiding questions:   - *Can the guidance be applied easily without the need to search for additional information?* - *Are the key constructs/procedures adequately operationalised?* | –/+/++ |  |
| Comprehensiveness  Guiding questions:   - *Does the guidance thoroughly describe information related to intervention adaptation in a new context?* - *Does the guidance thoroughly describe information related to intervention re-evaluation in a new context?^[[1]](#footnote-1)^* | –/+/++ |  |
| Relevance | | |
| Relevance for use with different interventions and by different stakeholders  Guiding question:   - *Can the guidance be applied to different types of population health interventions?* - *Can the guidance be adapted to the needs of different stakeholders (such as researchers, funders and users of evaluations)?* | –/+/++ |  |
| Legitimacy | | |
| Scientific basis and development process  Guiding questions:   - *Does the guidance describe its underlying theory and principles?* - *Does the guidance describe a rigorous development process (such as a comprehensive literature review and/or a consensus-based methodology)?* | –/+/++ |  |

Notes: –: “not at all”; +: “partially”; ++: “fully”.

1. Information on whether the guidance papers make recommendations on reporting intervention adaptation will also be documented and extracted. However, this will be separately from quality appraisal of the guidance papers as suggested in Table 2. [↑](#footnote-ref-1)
