# Supplementary material for: Adapting evidence-informed complex population health interventions for new contexts: a systematic review of guidance
Source: Implement Sci. 2019 Dec 17;14:105. doi: 10.1186/s13012-019-0956-5 (PMC6918624; doi:10.1186/s13012-019-0956-5)
Supplement: Supplementary file 4 — Additional file 4. Appraisal of the included guidance papers. This additional files provides the ratings of the included studies against the pre-defined quality appraisal criteria [file 13012_2019_956_MOESM4_ESM.docx]

**Additional File 3.**

**Table 1.** Appraisal of the included guidance papers

|  | **Practicality** | | | **Relevance** | **Legitimacy** |
| --- | --- | --- | --- | --- | --- |
|  | Understandability & clarity | Ease of use &  operationalisability | Comprehensiveness | Relevance for use by different stakeholders | Scientific basis & development process |
| Aarons (2012) | ++ | ++ | + | ++ | + |
| Aarons (2017) | ++ | + | + | ++ | + |
| Backer (2002) | ++ | + | + | + | + |
| Barrera (2006) | ++ | + | + | + | - |
| Bartholomew (2016) | + | ++ | + | ++ | + |
| Bernal (2006) | + | + | - | - | + |
| Card (2011) | ++ | ++ | + | ++ | + |
| Cardemil (2010) | ++ | ++ | ++ | + | - |
| Chen (2012) | ++ | ++ | + | + | + |
| Davidson (2013) | ++ | ++ | + | + | ++ |
| Domenech-Rodriguez (2005) | + | ++ | + | + | ++ |
| Goldstein (2012) | - | ++ | ++ | + | + |
| Hwang (2006 – 2009) | ++ | ++ | - | - | + |
| Kemp (2016) | ++ | ++ | ++ | + | + |
| Kilbourne (2007) | + | ++ | + | + | + |
| Kumpfer (2008 – 2016) | ++ | ++ | ++ | + | + |
| Lau (2006) | ++ | + | + | + | - |
| Lee (2008) | + | + | + | + | - |
| Maríñez-Lora (2016) | ++ | ++ | - | - | + |
| McKleroy (2006) | ++ | ++ | + | ++ | ++ |
| Nápoles (2013) | + | ++ | ++ | + | - |
| Nápoles (2018) | + | ++ | ++ | ++ | + |
| NCI RTIPs | + | + | + | + | - |
| Netto (2010) | + | + | + | + | + |
| Perez (2016) | ++ | ++ | - | + | + |
| Rolleri (2014) | ++ | ++ | + | + | + |
| Solomon (2006) | ++ | ++ | + | + | + |
| Sundell (2014) | ++ | ++ | + | + | - |
| Tomioka (2013) | - | + | + | + | - |
| Van Daele (2012) | ++ | ++ | + | + | + |
| Wang-Schweig (2014) | ++ | ++ | + | + | + |
| Wainberg (2007) | + | ++ | + | + | + |
| Wingood (2008) | + | ++ | + | + | + |
| Yong (2016) | + | + | - | + | + |

Notes: NCI RTIPs = National Cancer Institute Research-Tested Intervention Programmes.

| ++ | Addressed to a great extent |
| --- | --- |
| + | Addressed to some extent |
| - | Not addressed at all |
|  |  |
